# Human stem cell-derived ventral midbrain astrocytes exhibit a region-specific secretory profile

**DOI:** 10.1093/braincomms/fcad114

**Published:** 2023-04-17

**Authors:** Lucy A Crompton, Sarah F McComish, Tom G J Steward, Daniel J Whitcomb, Jon D Lane, Maeve A Caldwell

**Affiliations:** Department of Applied Sciences, Centre for Research in Biosciences, University of the West of England, Bristol BS16 1QY, UK; Cell Biology Laboratories, School of Biochemistry, University of Bristol, Bristol BS8 1TD, UK; Department of Physiology and Trinity College Institute for Neuroscience, Trinity College Dublin, Dublin 2, Ireland; Bristol Medical School, Faculty of Health Sciences, University of Bristol, Bristol BS1 3NY, UK; Bristol Medical School, Faculty of Health Sciences, University of Bristol, Bristol BS1 3NY, UK; Department of Applied Sciences, Centre for Research in Biosciences, University of the West of England, Bristol BS16 1QY, UK; Department of Physiology and Trinity College Institute for Neuroscience, Trinity College Dublin, Dublin 2, Ireland

## Abstract

This scientific commentary refers to ‘Human stem cell-derived astrocytes exhibit region-specific heterogeneity in their secretory profiles’, by Clarke *et al.* (https://doi.org/10.1093/brain/awaa258) in Brain.

**This scientific commentary refers to ‘Human stem cell-derived astrocytes exhibit region-specific heterogeneity in their secretory profiles’, by Clarke *et al*. (https://doi.org/10.1093/brain/awaa258) in Brain.**

It was recently reported in the journal *Brain* that human-induced pluripotent stem cell (hiPSC)-derived astrocytes display neuroinflammatory characteristics that correlate with their specific regional identities within the central nervous system (CNS).^1^ By generating hiPSC-derived dorsal forebrain and ventral spinal cord astrocytes, Clarke *et al*.^1^ demonstrated distinctive changes in protein secretion unique to each specific regional astrocyte population following inflammatory stimulation. These findings emphasize that, like neurons, the regional identity of astrocytes pre-determines their functional responses in the adult CNS, and crucially, that this emerging relationship between regional identity and functional idiosyncrasy also extends to the distinctive neuroinflammatory responses of astrocyte populations.^1^ Here, we present data that support and extend these observations, establishing that hiPSC-derived ventral midbrain astrocytes also demonstrate unique changes in their secretome in response to inflammation.

In Parkinson’s disease, neurodegeneration occurs in the substantia nigra, a structure within the ventral midbrain, where gradual loss of dopamine neurons leads to symptoms primarily associated with movement. As in many neurodegenerative conditions, neuroinflammation is a feature of Parkinson’s disease, and has recently been shown to pre-empt classic disease symptoms.^2^ Postmortem studies, as well as work in animal and *in vitro* models, have confirmed the pivotal nature of the relationship between astrocytes and microglia in the progression of neuroinflammation in Parkinson’s disease, and the crucial implications for this on the advancement of disease pathology.^3^ Therefore, as with other neurodegenerative conditions, it is essential that we appreciate the changing molecular relationships between astrocytes and neurons in areas of the brain that are primary sites of pathology in Parkinson’s disease, namely, the ventral midbrain.

In the healthy nervous system, astrocytes are neuroprotective, supporting neuronal function and survival.^4^ However, in a neuroinflammatory scenario, signals mediated by microglia induce astrocytic transformation to a reactive phenotype.^5^ This shift is characterized by increased cytokine secretion, transcriptional and morphological changes and alterations to astrocyte function (e.g. phagocytosis) to promote neuronal survival.^5^ In chronic neurodegenerative diseases such as amyotrophic lateral sclerosis and Parkinson’s disease, such protection is temporary, with ensuing chronic astrocyte reactivity, reduced neuronal support and ultimately, astrocyte-mediated neuronal toxicity.^3,5^ Astrocyte-secreted proteins amplify neuroinflammatory signaling, setting up a feedback loop promoting sustained neurodegeneration.^3,5^ It is hypothesized that these neurotoxic effects are a result of evolutionary antagonistic pleiotropism, where astrocytes evolved to provide protection against acute disease or injury, but are less well-equipped for post-reproduction stage protection in later life, a trait that is regrettably laid bare by the chronic nature of aging-related neurodegenerative diseases such as Parkinson’s disease.^4^

By recapitulating the signals required for differentiation of astrocytes in the embryonic ventral midbrain, and using methodologies in line with Clarke *et al*.,^1^ we have previously published a method for the generation of ventral midbrain astrocytes from hiPSCs^6^ (Fig. 1A). In brief, hiPSCs were exposed to dual SMAD inhibition through the addition of SB431542 (10 μM; Tocris) and LDN193189 (100 nM; Tocris) to induce neural fate, in combination with ventralizing factor Sonic hedgehog (SHH-C24ii; 200 ng/ml, Tocris) and the WNT activator CHIR99021 (0.8 μM; Tocris) required for midbrain induction.^6,7^ After 10 days, these factors were replaced with GDNF, BDNF (20 ng/mL; Peprotech) and ascorbic acid (200 μM; Merck), which support expansion of the resulting ventral midbrain neural progenitor population.^6,7^ From Day 30 onwards, the midbrain neural progenitor cells underwent extended expansion in culture under specific conditions to promote astrocyte fate (Fig. 1A(ii)). This was achieved by transferring cells into ASTRO media (Advanced DMEM/F12 + Glutamax, +2 × NEAA, + 1 × N2, + 0.1 × B27, all Thermo Fisher Scientific) containing human EGF and LIF (20 ng/mL; Peprotech). From Day 90 onwards, mature ventral midbrain astrocytes were generated by exposure to human BMP and LIF (20 ng/mL; Peprotech/Proteintech) for 10 days. BMP4 and LIF are required for the generation of mature astrocytes *in vivo* and *in vitro.*^10,11^ The hiPSC line NAS2 used in this study was kindly provided by Professor Tilo Kunath, University of Edinburgh.^12^

**Figure 1:**
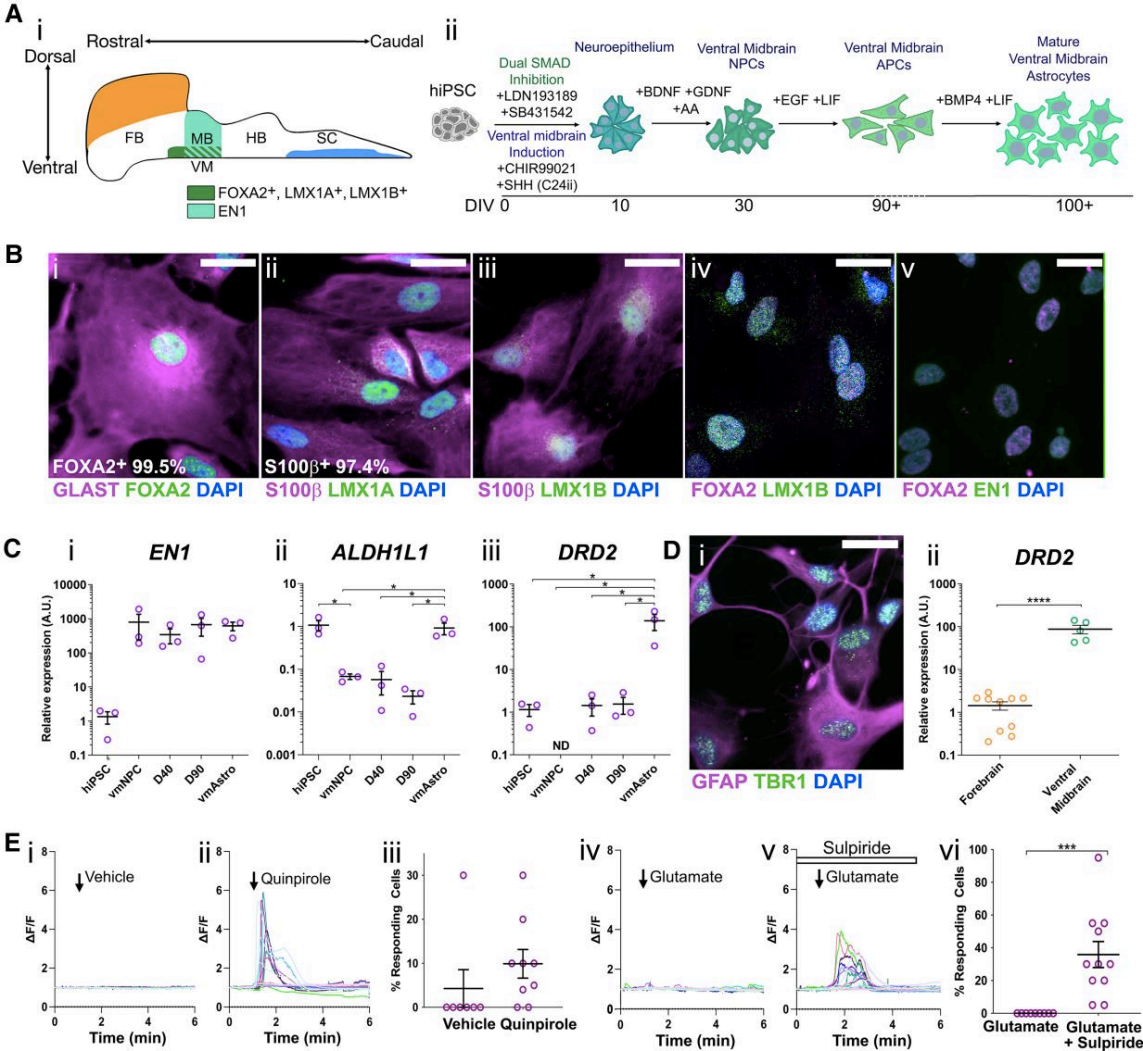
**Generation of functional, mature ventral midbrain astrocytes from hiPSCs.** (**A** (**i**)) Schematic modified from Clarke *et al*.^1^ of the regional patterning of the developing nervous system in relation to the body axes. Ventral midbrain identity is indicated by co-expression of ventrally expressed transcription factors FOXA2, LMX1A and LMX1B with midbrain specific transcription factor EN1, shown here in relation to the dorsal forebrain and ventral spinal cord. FB, forebrain; MB, midbrain; HB, hindbrain; VM, ventral midbrain; SC, spinal cord. (**A** (**ii**)) Overview of the differentiation of hiPSCs into ventral midbrain astrocytes. Ventral midbrain identity was induced through dual SMAD inhibition using SB431542 (10 μM) and LDN193189 (100 nM), with co-ordinate exposure to regional patterning factors SHH-C24ii (200 ng/ml) and CHIR99021 (0.8 μM) for 10 days, generating a progenitor population.^6–9^ After 10 days, these factors were replaced with GDNF, BDNF (20 ng/mL) and ascorbic acid (200 μM) which supported maintenance of the progenitors. From Day 30, cells were expanded in media containing human EGF and LIF (20 ng/mL), for a minimum 60 days of subsequent culture. From Day 90 onwards, mature ventral midbrain astrocytes were generated by exposure to human BMP and LIF (20 ng/mL) for 10 days.^6^ DIV, days in vitro; NPC, neural progenitor cell; APC, astrocyte progenitor cell. (**B**) Immunofluorescence on mature hiPSC-derived ventral midbrain astrocytes demonstrated expression of astrocyte markers GLAST and S100β, ventrally expressed transcription factors FOXA2, LMX1A and LMX1B and the midbrain expressed transcription factor EN1. Astrocytes were co-stained with nuclear stain DAPI. 99.5 ± 2.3% cells expressed FOXA2 and 97.4 ± 0.43% expressed S100β (*n* = 3, SEM). Scale bar = 25 μm. (**C**) Quantitative PCR demonstrated expression of midbrain marker *EN1* (**i**), mature astrocyte marker *ALDH1L1* (**ii**) and midbrain enriched, mature astrocyte marker *DRD2* (**iii**). 2^−ΔΔCt^ method of quantification, represented as relative expression, *n* = 3. One-way ANOVA, Tukey test for multiple comparisons. *P ≤ 0.05. AU, arbitrary units. (**D**) We generated forebrain astrocytes from hiPSCs, indicated by co-expression of GFAP and forebrain marker TBR1 (**i**). Quantitative PCR demonstrated the ventral midbrain enriched gene *DRD2* is specifically upregulated in ventral midbrain astrocytes, compared to their forebrain counterparts (**ii**) 2^−ΔΔCt^ method of quantification, represented as relative expression, *n* = 10 (forebrain), 5 (ventral midbrain). Unpaired *t*-test. ****P ≤ 0.0001. (**E**) Calcium imaging of ventral midbrain astrocytes quantifying changes in Fluo4-AM signal: example traces of cells with (**i**) vehicle alone; (**ii**) in the presence of quinpirole; (**iv**) in response to glutamate alone (no responses, *n* = 9 coverslips) and (**v**) with glutamate in the presence of sulpiride (responsive cells = 35.91% ± 7.94, *n* = 11 coverslips). Ventral midbrain astrocyte responsiveness to (**iii**) quinpirole, and (**vi**) to glutamate in the absence and presence of sulpiride (unpaired *t*-test. ***P ≤ 0.001).

Ventral midbrain astrocyte identity was confirmed by co-expression of ventral midbrain expressed transcription factors FOXA2,^13^ LMX1A^14^ and LMX1B^14^ and the midbrain transcription factor Engrailed-1 (EN1)^15,16^ with astrocyte markers (GLAST, S100β) (Fig. 1B and Supplementary Figs 1A–E). Quantification revealed that 99.5 ± 2.3% cells expressed FOXA2 (*n* = 3, SEM), and 97.4 ± 0.43% cells expressed S100β, confirming the high efficiency of our protocol to generate ventral midbrain astrocytes.

We used quantitative PCR to profile the temporal acquisition of ventral midbrain astrocyte identity throughout the protocol. Midbrain identity was induced at the earliest stages of the differentiation protocol as confirmed by upregulation of midbrain marker *EN1* (Fig. 1C(i)), indicative of accurate rostrocaudal patterning. This confirms our previous findings demonstrating robust ventral midbrain differentiation under these specific conditions.^6–8^ Expression of *EN1* was maintained in the ventral midbrain astroglial progenitors and in the mature astrocytes, indicating that ventral midbrain astrocytes retain regional identity (Fig. 1C(i)). Maturity of the astrocytes was confirmed by upregulation of the mature astrocyte marker, *ALDH1L1*, following exposure to BMP4 and LIF (Fig. 1C(ii)). The dopamine receptor D2 gene (*DRD2*) was also upregulated upon maturation (Fig. 1C(iii)). Its protein product, DRD2, is expressed at high levels in the substantia nigra and is critical both for dopaminergic signaling and for astrocyte neuroinflammatory modulation in the adult rodent ventral midbrain.^17,18^ For comparison, we generated forebrain astrocytes from hiPSCs using the methodology described but in the absence of patterning molecules SHH and CHIR99021. Forebrain astrocyte identity was confirmed by expression of astrocyte marker GFAP and forebrain specific transcription factor TBR1 (Fig. 1D(i) and Supplementary Fig. 1F). As expected, *DRD2* expression was highly enriched in ventral midbrain astrocytes, thus confirming the specificity of ventral midbrain astrocyte identity induced using the described protocol (Fig. 1D(ii)).

Dopaminergic signaling capacity of the hiPSC-derived ventral midbrain astrocytes was confirmed by calcium imaging, demonstrating responsiveness to the dopamine receptor agonist quinpirole (Fig. 1E(i–iii)). Ventral midbrain astrocytes were responsive to the neurotransmitter glutamate only when dopaminergic signaling was inhibited by the dopamine receptor antagonist sulpiride (Fig. 1E(i–iii)). This recapitulates findings by Xin *et al*.^17^ in astrocytes in the adult rodent midbrain. These data are consistent with a mature ventral midbrain astrocyte phenotype, thus suggesting our methods generate a comprehensive experimental model of astrocytes in the adult ventral midbrain. Therefore, we employed this model to investigate responses to neuroinflammation, and how these compared to those recorded in dorsal forebrain and ventral spinal cord astrocytes.^1^

We exposed the ventral midbrain astrocytes to the same combination of inflammatory stimuli as used in Clarke *et al*.,^1^ specifically, TNFα/IL1α/C1q. We also treated with IL1β and IL6 as these cytokines are elevated in Parkinson’s disease.^5,19–21^ The reactive transformation was confirmed following TNFα/IL1α/C1q and IL1β treatments by upregulation of genes known to be associated with the reactive state,^5,19^ but IL6 treatment was ineffective (Fig. 2A). We therefore selected TNFα/IL1α/C1q and IL1β exposures for further investigation into the reactive astrocyte phenotype.

**Figure 2:**
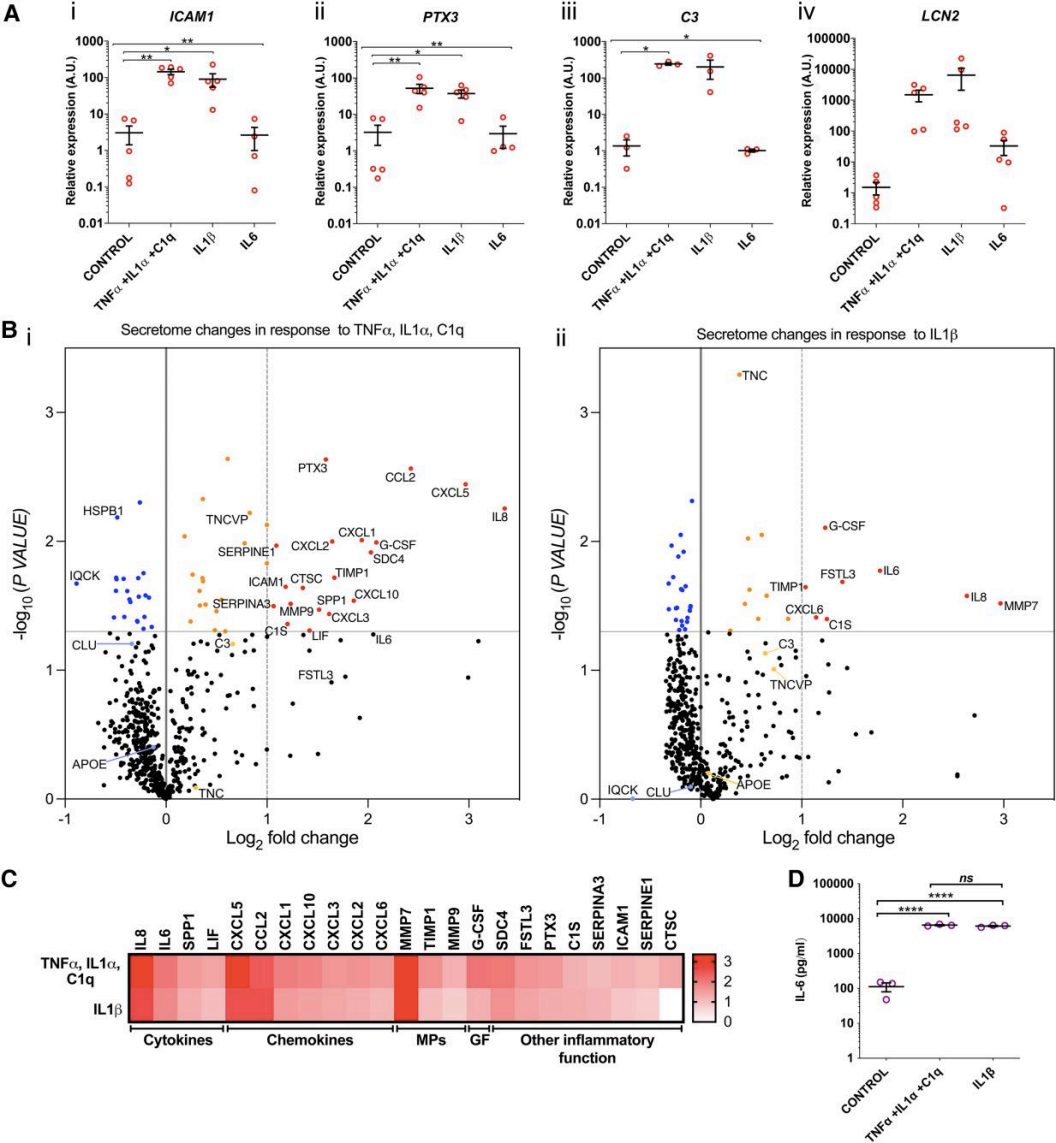
**Reactive hiPSC-derived ventral midbrain astrocytes demonstrate a characteristic neuroinflammatory protein secretion profile.** (**A**) Quantitative PCR demonstrates upregulation of the following markers of astrocyte reactivity in response to 24 h exposure to either TNFα (30 ng/mL), IL1α (3 ng/mL), C1q (400 ng/mL) or IL1β (3 ng/mL) or IL6 (250 ng/mL): (i) *ICAM2*; (ii) *PTX3*; (iii) *C3*; (iv) *LCN2*. Exposure to IL6 did not result in increases in these markers. 2^−ΔΔCt^ method of quantification, represented as relative expression, *n* = 5 (*C3*, *n* = 3). One-way ANOVA, Dunnett test for comparison to control. *P ≤ 0.05, **P ≤ 0.01. AU, arbitrary units. (**B**) Tandem mass tag (TMT) analysis of astrocyte conditioned media in response to (i) TNFα, IL1α, C1q; (ii) IL1β. *N* = 4. Proteins that increased above 2-fold (threshold indicated by the dashed line) are represented in red, *P* < 0.05; proteins that increased less than 2-fold, are represented in orange, *P* < 0.05; proteins that decreased less that 2-fold are represented in blue, *P < 0.05. (**C**) Exposure to either TNFα, IL1α, C1q or IL1β results in increased secretion of a profile of proteins including cytokines, chemokines, metalloproteinases (MPs), growth factors (GF) and a number of proteins which have ascribed functions relating to inflammation. Listed proteins had over a 1-fold change and *P < 0.05 in one of both treatments. *n* = 4. Unpaired *t*-test. (**D**) Validation by ELISA demonstrates significantly increased IL6 secretion in response to TNFα, IL1α, C1q or IL1β. *n* = 3. One-way ANOVA, ****P < 0.0001.

To investigate how their regional identity influences the reactive phenotype, we carried out secretome analysis of hiPSC-derived ventral midbrain astrocytes using tandem mass tag (TMT) spectroscopy to compare astrocyte conditioned media after 24 h in the absence or presence of neuroinflammatory stimulants (Fig. 2B(i, ii)). Analysis of the TNFα, IL1α, C1q and IL1β treated populations revealed increased secretion of proteins encoded by genes previously described as unique markers of human astrocyte reactivity,^5,19^ namely, PTX3, SERPINA3, ICAM1 and C3 (Fig. 2B). This further validated the accuracy of our ventral midbrain astrocytes in recapitulating the characteristics of endogenous astrocytes in neuroinflammation.

Detailed secretome analysis revealed some commonalities in the proteins secreted by ventral midbrain astrocytes (Fig. 2B and C), when compared with dorsal forebrain and ventral spinal cord astrocytes.^1^ It was striking that treatment with either TNFα/IL1α/C1q, or IL1β, generated similar changes in protein secretion by ventral midbrain astrocytes (Fig. 2B and C). Like their dorsal forebrain and spinal cord counterparts, when treated with TNFα/IL1α/C1q (or indeed with IL1β), ventral midbrain astrocytes demonstrated increased secretion of the cytokines IL8 and IL6, the chemokines CCL2, CXCL1, CXCL10 and CCL5, the metalloprotease MMP9 and the growth factor G-CSF (Fig. 2C). These proteins may represent a universal neuroinflammatory astrocyte signature. We also saw large increases in secretion of the chemokine CCL2 and the metalloprotease TIMP1 (Fig. 2C). By contrast, Clarke *et al*.^1^ reported no changes in levels of these specific proteins in either of the regional astrocyte populations examined, suggesting that CCL2 and TIMP1 characterize the unique ventral midbrain neuroinflammatory astrocyte secretome. Clarke *et al*.^1^ highlighted elevated secretion of IL1α and IL6 with respect to astrocyte-mediated inflammation, which was more pronounced in dorsal forebrain reactive astrocytes than in spinal cord reactive astrocytes. However, our secretome data revealed increased IL6 secretion without accompanying IL1α secretion in reactive ventral midbrain astrocytes (Fig. 2B and C). We provide validation of this result by ELISA, which demonstrated the same increase in IL6 secretion by reactive ventral midbrain astrocytes, but the complete absence of IL1α secretion (Fig. 2D; endogenous IL1α negligible by ELISA—data not shown). The differences in our data when compared to that of Clarke *et al*.^1^ demonstrate the unique neuroinflammatory characteristics of the ventral midbrain class of astrocytes in this experimental setting.

We also recorded increased secretion of a unique array of additional proteins by reactive ventral midbrain astrocytes, that were not secreted by forebrain or ventral spinal cord astrocytes.^1^ These included the neuromodulatory cytokines SPP1 (also known as OSTEOPONTIN) and LIF, the chemokines CXCL2, CXCL3, CXCL5 and CXCL6 and the metalloproteinase MMP7 (Fig. 2C). SPP1 is particularly interesting in the context of neuroinflammation in Parkinson’s disease, as this cytokine is elevated in the brains of Parkinson’s disease patients and has a direct impact on the survival of dopaminergic neurons.^22^ In addition, metalloproteinases (MMP-7, TIMP-1 and MMP-9) have multifaceted roles in neuroinflammation, neuronal survival and neurogenesis.^23^

A further observation relating to the regional identity of the hiPSC-derived ventral midbrain astrocytes emerged when comparing with data in a recent paper by Kostuk *et al*.^24^ that characterized the transcriptome of adult mouse astrocytes from two subregions of the ventral midbrain: the substantia nigra and the ventral tegmental area. Several of the most abundant proteins present in the secretome of our hiPSC-derived ventral midbrain astrocytes are encoded by genes that Kostuk *et al*.^24^ identified as enriched specifically in astrocytes of the substantia nigra [*Col1a1*, *Tnc* (encodes TNC and TNCVP) and *Postn*] (Supplementary Table 1). Interestingly, *TNC* is also expressed at high levels in astrocytes isolated from postmortem samples of the human substantia nigra.^25^ However, we also recorded numerous secreted proteins that were associated with the ventral tegmental area in mouse in the Kostuk *et al*.^24^ study (CCL2, IL6, GREM1, CXCL1 and TGFB2) (Supplementary Table 1). This suggests a degree of subregional heterogeneity in our hiPSC-derived ventral midbrain astrocyte.

Our data revealed a number of proteins demonstrating minor reductions in secretion in response to TNFα/IL1α/C1q or IL1β; however, we saw little conservation in these changes across the two treatment groups. Of potential interest, one of these was the heat shock protein HSPB1, which was reduced in response to TNFα/IL1α/C1q. HSPB1 is highly expressed in astrocytes, is an established regulator of neuroinflammation, as well as demonstrating changes in expression related to both aging and neurodegenerative diseases, including Parkinson’s disease.^26^ Also reduced in the secretome of TNFα/IL1α/C1q treated ventral midbrain astrocytes were neurodegeneration-implicated proteins, CLU, APOE (minimal reduction) and IQCK^27,28^ (Fig. 2B). These proteins are interesting candidates for further investigation in relation to astrocyte-mediated neuroinflammation in disease progression. For instance, Guttenplan *et al*. (2021) recently demonstrated that mouse CLU mediates neurotoxicity of reactive rat astrocytes alongside APOE through association with specific lipids.^29^

Together these findings highlight how astrocyte-mediated changes in the brain can play pivotal roles in the progression of neurodegeneration in Parkinson’s disease, underscoring the importance of considering astrocyte regional functional identity for understanding localized diseases such as Parkinson’s disease and amyotrophic lateral sclerosis. Crucially, it is clear that astrocyte reactivity is characterized by regionally encoded changes in protein secretion profiles (Fig. 2C), so understanding the molecular basis of such regionally encoded functional heterogeneity in astrocyte populations will be essential for the development of future neuromodulatory clinical strategies in diseases including Parkinson’s disease and amyotrophic lateral sclerosis.^1^

## Supplementary Material

fcad114_Supplementary_Data

## Data Availability

Data supporting the findings of this study are available within the article or are available in the Supplementary material.

## Supplementary material

See Supplementary material for additional information on materials and methods.

## References

[1] Clarke BE , Taha DM, Ziff OJ, et al Human stem cell-derived astrocytes exhibit region-specific heterogeneity in their secretory profiles. Brain. 2020;143(10):e85. 32895697 10.1093/brain/awaa258 PMC7586081

[2] Stokholm MG , Iranzo A, Ostergaard K, et al Assessment of neuroinflammation in patients with idiopathic rapid-eye-movement sleep behaviour disorder: A case-control study. Lancet Neurol. 2017;16(10):789–796. 28684245 10.1016/S1474-4422(17)30173-4

[3] Yun SP , Kam TI, Panicker N, et al Block of A1 astrocyte conversion by microglia is neuroprotective in models of Parkinson’s disease. Nat Med. 2018;24(7):931–938. 29892066 10.1038/s41591-018-0051-5 PMC6039259

[4] Crompton LA , Cordero-Llana O, Caldwell MA. Astrocytes in a dish: Using pluripotent stem cells to model neurodegenerative and neurodevelopmental disorders. Brain Pathol. 2017;27(4):530–544. 28585380 10.1111/bpa.12522 PMC8028895

[5] Liddelow SA , Guttenplan KA, Clarke LE, et al Neurotoxic reactive astrocytes are induced by activated microglia. Nature. 2017;541(7638):481–487. 28099414 10.1038/nature21029 PMC5404890

[6] Crompton LA , McComish SF, Stathakos P, Cordero-Llana O, Lane JD, Caldwell MA. Efficient and scalable generation of human ventral midbrain astrocytes from human-induced pluripotent stem cells. J Vis Exp. 2021;2(176):e62095. 10.3791/62095 34661566

[7] Stathakos P , Jimenez-Moreno N, Crompton L, Nistor P, Caldwell MA, Lane JD. Imaging autophagy in hiPSC-derived midbrain dopaminergic neuronal cultures for Parkinson’s disease research. Methods Mol Biol. 2019;1880:257–280. 30610703 10.1007/978-1-4939-8873-0_17

[8] Stathakos P , Jiménez-Moreno N, Crompton LA, et al A monolayer hiPSC culture system for autophagy/mitophagy studies in human dopaminergic neurons. Autophagy. 2021;17(4):855–871. 32286126 10.1080/15548627.2020.1739441 PMC8078667

[9] Chambers SM , Fasano CA, Papapetrou EP, Tomishima M, Sadelain M, Studer L. Highly efficient neural conversion of human ES and iPS cells by dual inhibition of SMAD signaling. Nat Biotechnol. 2009;27(3):275–280. 19252484 10.1038/nbt.1529 PMC2756723

[10] Nakashima K , Wiese S, Yanagisawa M, et al Developmental requirement of gp130 signaling in neuronal survival and astrocyte differentiation. J Neurosci. 1999;19(13):5429–5434. 10377352 10.1523/JNEUROSCI.19-13-05429.1999 PMC6782325

[11] Nakashima K , Yanagisawa M, Arakawa H, Taga T. Astrocyte differentiation mediated by LIF in cooperation with BMP2. FEBS Lett. 1999;457(1):43–46. 10486560 10.1016/s0014-5793(99)00997-7

[12] Devine MJ , Ryten M, Vodicka P, et al Parkinson’s disease induced pluripotent stem cells with triplication of the alpha-synuclein locus. Nat Commun. 2011;2:440. 21863007 10.1038/ncomms1453 PMC3265381

[13] Ang SL , Wierda A, Wong D, et al The formation and maintenance of the definitive endoderm lineage in the mouse: Involvement of HNF3/forkhead proteins. Development. 1993;119(4):1301–1315. 8306889 10.1242/dev.119.4.1301

[14] Andersson E , Tryggvason U, Deng Q, et al Identification of intrinsic determinants of midbrain dopamine neurons. Cell. 2006;124(2):393–405. 16439212 10.1016/j.cell.2005.10.037

[15] Davis CA , Joyner AL. Expression patterns of the homeo box-containing genes En-1 and En-2 and the proto-oncogene int-1 diverge during mouse development. Genes Dev. 1988;2(12B):1736–1744. 2907320 10.1101/gad.2.12b.1736

[16] Kee N , Volakakis N, Kirkeby A, et al Single-cell analysis reveals a close relationship between differentiating dopamine and subthalamic nucleus neuronal lineages. Cell Stem Cell. 2017;20(1):29–40. 28094018 10.1016/j.stem.2016.10.003

[17] Xin W , Schuebel KE, Jair KW, et al Ventral midbrain astrocytes display unique physiological features and sensitivity to dopamine D2 receptor signaling. Neuropsychopharmacology. 2019;44(2):344–355. 30054584 10.1038/s41386-018-0151-4 PMC6300565

[18] Zhu J , Hu Z, Han X, et al Dopamine D2 receptor restricts astrocytic NLRP3 inflammasome activation via enhancing the interaction of beta-arrestin2 and NLRP3. Cell Death Differ. 2018;25(11):2037–2049. 29786071 10.1038/s41418-018-0127-2 PMC6219479

[19] Barbar L , Jain T, Zimmer M, et al CD49f is a novel marker of functional and reactive human iPSC-derived astrocytes. Neuron. 2020;107(3):436–453 e12. 32485136 10.1016/j.neuron.2020.05.014 PMC8274549

[20] Williams-Gray CH , Wijeyekoon R, Yarnall AJ, et al Serum immune markers and disease progression in an incident Parkinson’s disease cohort (ICICLE-PD). Mov Disord. 2016;31(7):995–1003. 26999434 10.1002/mds.26563 PMC4957620

[21] Garcia-Esparcia P , Llorens F, Carmona M, Ferrer I. Complex deregulation and expression of cytokines and mediators of the immune response in Parkinson’s disease brain is region dependent. Brain Pathol. 2014;24(6):584–598. 24593806 10.1111/bpa.12137 PMC8029304

[22] Maetzler W , Berg D, Schalamberidze N, et al Osteopontin is elevated in Parkinson’s disease and its absence leads to reduced neurodegeneration in the MPTP model. Neurobiol Dis. 2007;25(3):473–482. 17188882 10.1016/j.nbd.2006.10.020

[23] Behl T , Kaur G, Sehgal A, et al Multifaceted role of matrix metalloproteinases in neurodegenerative diseases: Pathophysiological and therapeutic perspectives. Int J Mol Sci. 2021;22(3):1413. 33573368 10.3390/ijms22031413 PMC7866808

[24] Kostuk EW , Cai J, Iacovitti L. Subregional differences in astrocytes underlie selective neurodegeneration or protection in Parkinson’s disease models in culture. Glia. 2019;67(8):1542–1557. 31025779 10.1002/glia.23627 PMC6594409

[25] Smajić S , Prada-Medina CA, Landoulsi Z, et al Single-cell sequencing of human midbrain reveals glial activation and a Parkinson-specific neuronal state. Brain. 2022;145(3):964–978. 34919646 10.1093/brain/awab446 PMC9050543

[26] Dukay B , Csoboz B, Tóth ME. Heat-shock proteins in neuroinflammation. Front Pharmacol. 2019;10:920. 31507418 10.3389/fphar.2019.00920 PMC6718606

[27] Smith AM , Davey K, Tsartsalis S, et al Diverse human astrocyte and microglial transcriptional responses to Alzheimer’s pathology. Acta Neuropathol. 2022;143(1):75–91. 34767070 10.1007/s00401-021-02372-6 PMC8732962

[28] Kunkle BW , Grenier-Boley B, Sims R, et al Genetic meta-analysis of diagnosed Alzheimer's disease identifies new risk loci and implicates Aβ, tau, immunity and lipid processing. Nat Genet. 2019;51(3):414–430. 30820047 10.1038/s41588-019-0358-2 PMC6463297

[29] Guttenplan KA , Weigel MK, Prakash P, et al Neurotoxic reactive astrocytes induce cell death via saturated lipids. Nature. 2021;599(7883):102–107. 34616039 10.1038/s41586-021-03960-y PMC12054010

