# Supplementary material for: Human stem cell-derived ventral midbrain astrocytes exhibit a region-specific secretory profile
Source: Brain Commun. 2023 Apr 17;5(2):fcad114. doi: 10.1093/braincomms/fcad114 (PMC10146926; doi:10.1093/braincomms/fcad114)
Supplement: fcad114_Supplementary_Data [file fcad114_supplementary_data.zip › Supplementary material.pdf]

Supplementary materials and methods

Immunocytochemistry. Astrocytes were seeded on Geltrex coated glass coverslips, were fixed in 4% formaldehyde (10 minutes at room temperature) and blocked/permealized with 10% horse serum/1% BSA in PBS + 0.1% Triton-X (PBTx). Primary antibodies: anti-GLAST (20785-1-AP, Proteintech); anti-GFAP (Z03342, Dako or ab4674, Abcam); anti-FOXA2 (sc-101060, SCBT); anti-LMX1A (AB10533, Merck-Millipore); anti-LMX1B (18278-1, Proteintech); anti-S100β (SAB4200671, Merck); anti-EN1 (GTX134013, Stratech); anti-TBR1 (ab31940, Abcam). Secondary antibodies: AlexaFluor conjugated goat anti-mouse, goat anti-rabbit or goat anti-chicken (ThermoFisher Scientific). Images were collected using either a SP8 Leica confocal microscope or an Olympus BX53 widefield microscope.

For quantification of FOXA2 and S100β positive cells, images were collected form 2 coverslips per independent experimental repeat (N=3) and percentage of cells positive for these markers were quantified in 4 fields of view per coverslip.

qRT-PCR gene expression analysis. Total RNA was extracted using a RNAeasy Mini Kit (Qiagen). Reverse transcription was carried out using the Taqman reverse transcription reagents (ThermoFisher Scientific). Taqman gene expression assays were used for PCR quantification according to manufacturer's specifications (*ALDH1L1*; *DRD2*; *EN1*; *C3*; *LCN2*; *ICAM1; PTX3* (ThermoFisher Scientific)). qRT-PCR was carried out on each sample in triplicate.

Calcium Imaging. Astrocytes were plated on 15 mm coverslips. Prior to imaging, coverslips were washed with HEPES-buffered saline (HBS) and incubated with 5 mM Fluo4-AM (AAT Bioquest) in HEPES with 1% BSA for 1hr (37°C, 5% CO_2, 20% O_2). Coverslips were transferred into HBS at room temperature for 30 min to allow for de-esterification. Coverslips were transferred to a RC-42LP recording chamber (Warner Instruments), housed on a Leica DM IRB inverted microscope and constantly perfused with HBS (2-3 ml/min). Time-lapse images were acquired at 0.5Hz over 6 min. Images were captured using a Hamamatsu Orca Spark CMOS camera with MicroManager software (version 2.0gamma) [1]. Test drugs were applied via a gravity-fed perfusion line, in 2 ml of HBS solution. Images were analysed using FIJI/ImageJ (version 1.52e)[2,3]. Regions of interest (ROIs) were drawn over 20 pseudo-randomly chosen cells, distributed over the field of view. Mean intensity of each ROI was measured across the 6 min recording (720 frames). Following background subtraction, baseline mean intensities were calculated for each ROI over the first 20 frames. All values were then plotted as a relative to baseline. A cell was considered responsive if it displayed a calcium event with a peak greater than 1.5 of baseline, occurring within 60 frames of drug perfusion.

Inflammatory stimulation of ventral midbrain astrocytes. Ventral midbrain astrocytes were seeded at a density of 65,000/cm^2 on 0.25x Geltrex coated plasticware in minimal media (1:1000 N2, DMEM:F12 + Glutamax) and left at 37°C, 5% CO2 in a humidified incubator for 24hr. After 24hr media, astrocytes were washed with minimal media and this was replaced with fresh minimal media containing TNFα (30 ng/ml), IL1α (3 ng/ml), C1q (400 ng/ml) or IL1β (3 ng/ml) or IL6 (250 ng/ml), or minimal media only and replaced at 37°C, 5% CO_2 in a humidified incubator for 24hr.

Generation of conditioned media for secretome analysis. Ventral midbrain astrocytes were seeded at a density of 130,000/cm^2 on 0.1x Geltrex coated 6-well plate in minimal media (1:1000 N2, DMEM:F12 + Glutamax (phenol red free) and left at 37°C, 5% CO2 in a humidified incubator. After 24 hours media, astrocytes were washed with fresh minimal media containing TNFα (30 ng/ml), IL1α (3 ng/ml), C1q (400 ng/ml) or IL1β (3 ng/ml), or minimal media only and replaced at 37°C, 5% CO_2 in a humidified incubator for 24hr after which media was collected for secretome analysis.

Secretome analysis of astrocyte conditioned media using TMT labelling and high pH reversed-phase chromatography. Astrocyte conditioned media was generated from astrocytes differentiated from the hiPSC line NAS2[4]. Media was centrifuged at 500 xg to remove debris, snap frozen and stored at -80°C, and thawed on ice. Conditioned media was prepared from 4 independent experiments (N=4) in the presence of inflammatory stimulation (described previously) or under control conditions, excluding inflammatory factors. Total protein content of conditioned media samples was determined by BCA assay (ThermoFisher Scientific). Aliquots equivalent to 30 µg were digested with trypsin (1.25 µg trypsin; 37°C, overnight), labelled with Tandem Mass Tag (TMT) ten plex reagents according to the manufacturer's protocol (ThermoFisher Scientific) and the labelled samples pooled. Pooled samples were desalted using a SepPak cartridge according to the manufacturer's instructions (Waters). Eluates were evaporated to dryness, resuspended in 20 mM ammonium hydroxide, pH 10, and prior to fractionated, subjected to high pH reversed-phase chromatography using an Ultimate 3000 liquid chromatography system in line with an Orbitrap Fusion Lumos mass spectrometer (ThermoFisher Scientific). All spectra were acquired using an Orbitrap Fusion Lumos mass spectrometer controlled by Xcalibur 3.0 software (ThermoFisher Scientific) and operated in data-dependent acquisition mode using an SPS-MS3 workflow. Raw data files were processed and quantified using Proteome Discoverer software v2.1 (ThermoFisher Scientific) and searched against the UniProt Human database using the SEQUEST HT algorithm. Peptide precursor mass tolerance was set at 10ppm, and MS/MS tolerance was set at 0.6Da. Raw data was processed in R and exported to excel. The log2 data and a Welch's t-test were performed.

Statistical analysis. All quantitative data are represented as means ± standard error of the means (SEMs). Statistical significance testing was performed using appropriate tests as detailed in figure legends (one-way ANOVA with Tukeys's or Dunnett's multiple comparisons test multiple comparisons test for three or greater groups; unpaired *t*-test for comparison of two groups. Analysis using GraphPad Prism 9.2 (GraphPad, USA).

Supplementary figure legends

Figure S1. Immunocytochemistry images represented in single colour channels.

[A-E] Immunofluorescence on mature hiPSC-derived ventral midbrain astrocytes demonstrated co-expression of ventral midbrain transcription factor FOXA2 and mature astrocyte marker GLAST (A; corresponds to Fig. 1Bi), ventral midbrain expressed transcription factors LMX1A and LMX1B and astrocyte marker S100β (B, C; corresponds to Fig. 1Bii and iii). LMX1A and LMX1B also demonstrated co-expression with ventral midbrain marker FOXA2 (D; corresponds to Fig. 1Biv). Midbrain specific transcription factor EN1 is co-expressed in the mature astrocytes with ventral marker FOXA2 (E; corresponds to Fig. 1Bv). Forebrain astrocytes generated in parallel co-express forebrain transcription factor TBR1 with astrocyte specific protein GFAP (F; corresponds to Fig. 1Di). Scale bar = 25 µm.

References

1. Edelstein A, Amodaj N, Hoover K, Vale R, Stuurman N. Computer control of microscopes using µManager. *Curr Protoc Mol Biol*. Oct 2010;Chapter 14:Unit14.20. doi:10.1002/0471142727.mb1420s92

2. Schindelin J, Arganda-Carreras I, Frise E, et al. Fiji: an open-source platform for biological-image analysis. *Nat Methods*. Jun 28 2012;9(7):676-82. doi:10.1038/nmeth.2019
3. Schneider CA, Rasband WS, Eliceiri KW. NIH Image to ImageJ: 25 years of image analysis. *Nat Methods*. Jul 2012;9(7):671-5. doi:10.1038/nmeth.2089
4. Devine MJ, Ryten M, Vodicka P, et al. Parkinson's disease induced pluripotent stem cells with triplication of the alpha-synuclein locus. *Nat Commun*. 2011;2:440.

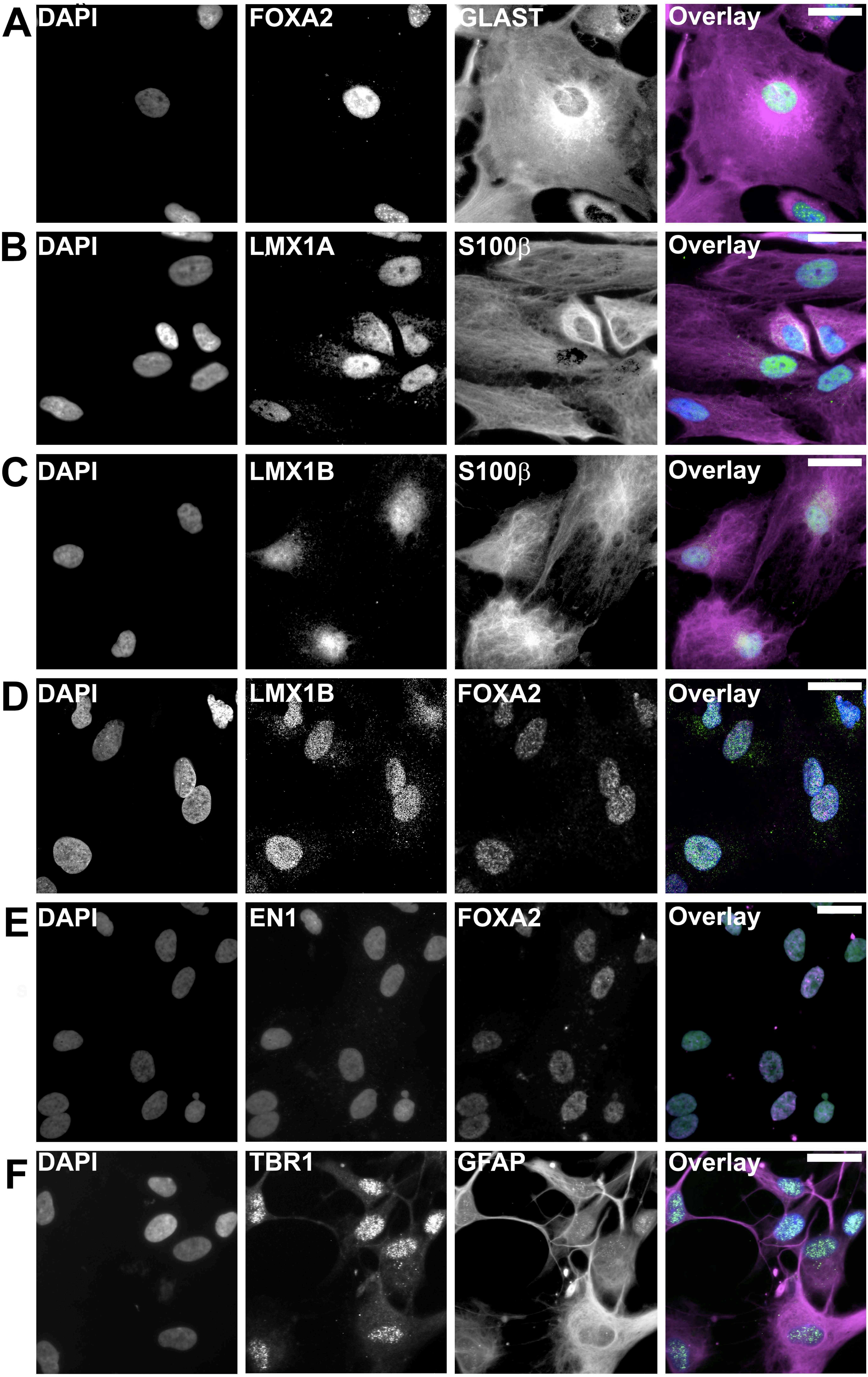
A
DAPI
FOXA2
GLAST
Overlay
B
DAPI
LMX1A
S100β
Overlay
C
DAPI
LMX1B
S100β
Overlay
D
DAPI
LMX1B
FOXA2
Overlay
E
DAPI
EN1
FOXA2
Overlay
F
DAPI
TBR1
GFAP
Overlay
